# De novo assembly of bacterial genomes with repetitive DNA regions by dnaasm application

**DOI:** 10.1186/s12859-018-2281-4

**Published:** 2018-07-18

**Authors:** Wiktor Kuśmirek, Robert Nowak

**Affiliations:** Institute of Computer Science, Warsaw University of Technology, Nowowiejska 15/19, Warsaw, 00-665 Poland

**Keywords:** De novo assembling, De Bruijn graph, Next generation sequencing, Tandem repeats

## Abstract

**Background:**

Many organisms, in particular bacteria, contain repetitive DNA fragments called tandem repeats. These structures are restored by DNA assemblers by mapping paired-end tags to unitigs, estimating the distance between them and filling the gap with the specified DNA motif, which could be repeated many times. However, some of the tandem repeats are longer than the distance between the paired-end tags.

**Results:**

We present a new algorithm for de novo DNA assembly, which uses the relative frequency of reads to properly restore tandem repeats. The main advantage of the presented algorithm is that long tandem repeats, which are much longer than maximum reads length and the insert size of paired-end tags can be properly restored. Moreover, repetitive DNA regions covered only by single-read sequencing data could also be restored. Other existing de novo DNA assemblers fail in such cases.

The presented application is composed of several steps, including: (i) building the de Bruijn graph, (ii) correcting the de Bruijn graph, (iii) normalizing edge weights, and (iv) generating the output set of DNA sequences.

We tested our approach on real data sets of bacterial organisms.

**Conclusions:**

The software library, console application and web application were developed. Web application was developed in client-server architecture, where web-browser is used to communicate with end-user and algorithms are implemented in C++ and Python. The presented approach enables proper reconstruction of tandem repeats, which are longer than the insert size of paired-end tags. The application is freely available to all users under GNU Library or Lesser General Public License version 3.0 (LGPLv3).

## Background

Next-generation sequencing (NGS) has dramatically reduced the time and the cost of producing genome sequences using massively parallel technologies [1]; therefore, we observe exponential increase of sequencing data [2]. The reduction of cost and sequencing the time allowed to develop many applications, such as biosurveillance, bioforensics, and infectious disease epidemiology [3]. What is more, genome-scale metabolic modeling and metagenomic sequencing of patient samples could improve the efficiency of diagnosis and treatment of diseases in the near future. All of the shown above practical applications are based largely on the genome sequencing of bacterial organisms.

The sequencing procedure for bacterial organisms has changed a lot over the last 20 years. In 1995, the first two sequenced bacterial organisms were published. Over time, sequencing technology has evolved, and now bacterial sequencing has become the standard procedure. However, many of the sequenced bacterial genomes are currently incomplete - for example 90% of bacterial genomes in GenBank [3] are incomplete. In many cases the incompleteness is a result of the occurrence of repetitive sequences in bacterial genomes that can not always be reconstructed from short DNA reads from second-generation sequencing.

Some of the repetitive DNA regions could represent a structure called tandem repeat - a sequence built from several identical DNA fragments lying one after another, caused mainly by strand-slippage replication [4]. Bacterial genomes contain up to several dozens of tandem repeats divided into two groups: intragenic and intergenic. Nevertheless, only a small number of tandem repeats have been functionally studied to date; for example, some of the functions of specific genes can be modulated by instability of tandem repeats. This process allows bacteria adaptation to a new environment in a short term without complicated mutation [5].

Current DNA assemblers, like ABySS [6], Velvet [7] or SPAdes [8], reconstruct tandem repeats using the information contained in paired-end tags. However, some repetitive regions may be much longer than maximum reads length and the insert size of paired-end tags. Such regions cannot be reconstructed by modern DNA assemblers.

Here, we present a new algorithm for DNA assembly, which uses the relative frequency of DNA reads to properly reconstruct tandem repeats. The main advantage of our approach is that tandem repeats, which are longer than the insert size of paired-end tags, can also be properly reconstructed, while other de novo genome assemblers fail in such cases. What is more, long tandem repeats could also be restored if only single-read sequencing data is available. The presented approach requires high sequencing coverage, currently easily achievable for bacterial genomes, but the tandem repeats reconstruction process could significantly improve contiguity over previous approaches, which was also indicated in the study.

## Implementation

In this section, we present the main data processing pipeline that has been implemented in a new DNA assembler named ’dnaasm’. We use de Bruijn graph due to its efficiency for the next generation sequencing data. We mainly focus on describing the process of estimation tandem repeats length and the process of reconstruction repetitive DNA fragments. We also present the main implementation aspects that make our application memory and computing efficient.

### Assembly workflow

#### Building and correcting de Bruijn graph

The first stage of de novo assembling in ’dnaasm’ is de Bruijn graph construction. As in the typical de novo DNA assembler, dnaasm builds de Bruijn graph from input set of DNA reads by splitting each read into set of k-mers. Each k-mer represents a substring of length *k* from input DNA read - a number of k-mers generated from single DNA read of length *L* is equal to *L*−*k*+1. Then, on the constructed de Bruijn graph, some algorithms for error correction are applied, similar to algorithms implemented previously [7]. Especially, dnaasm uses algorithms for removing tips, bubbles and edges of low weight. At this stage, all edges representing DNA sequencing errors should be removed from the de Bruijn graph. Moreover, the edges of the de Bruijn graph represent substrings of length *k* and in the presented approach each edge has an additional property, the integer number named edge weight, which depicts a number of occurrence of DNA fragment of length *k* in the input set of DNA reads, as in A-Bruijn graph [9].

The specified edge weight *w* is equal to exact k-mer count, where edge represents specified DNA substring of length *k* in the set of reads. Let’s consider ideal assembler input *R*, called k-spectrum, where reads are generated without errors from a circular bacterial genome of sequence *s*_0_*s*_1_...*s*_*G*−1_ of length *G*, and reads *r*∈*R* have identical length *L*, and *R* is a set of all substrings *s*_*i*_...*s*_*i*+*k*−1_ for 0≤*i*≤*G*. The edge weight *w* in this case is $w = \frac {N(L-k+1)}{G}$ for non-repetitive k-mers, where *N* depicts a number of reads, and edge weight $w = \frac {\Delta }{d} \frac {N(L-k+1)}{G}$, for k-mers inside tandem repeat of length *n*, where repetitive motif of length *d* is repeated ⌊*n*/*d*⌋ times (integer division), tandem repeat is longer than graph dimension, *n*>*k*, and *Δ*=*n*−*k*+1.

We prove in [10] the edge weight *w* for de Bruijn graph of dimension *k*, for error-less set of *N* reads with identical length *L*, assuming uniform distribution of the reads position over input circular bacterial genome of length *G*, is a random variable with Poisson distribution (probability mass function is $P\left (x \right) = \frac {{e^{- \lambda } \lambda ^{x} }}{{x!}}$), as depicted in Eq. 1.

1 $$ W \!\sim\! {Poisson}~({\lambda})~\text{where}~\lambda \,=\, \frac{NL(L-k+1)\Delta}{Gkd}, \Delta = n-k+1,  $$

#### Estimating a number of repeats

After the de Bruijn graph construction and correction, dnaasm application estimates the number of occurrences of a given DNA fragment, represented by the edge in the de Bruijn graph, in the investigated genome. This process consists of two stages: firstly, the normalization factor is calculated in accordance with the equation:

2 $$ p=\frac{G}{N(L-k+1)}  $$

The presented normalization factor is the result of modeling edge weight by Poisson distribution described in Eq. 1. Then, the edge normalization is carried out - it consists in multiplying the input edge weight (which is the number of occurrences of the DNA fragment represented by the edge in the input set of DNA reads) by the previously calculated normalization factor. The multiplication result is rounded to the nearest integer, which represents the number of occurrences of the DNA fragment represented by the edge in the investigated genome. This step could be briefly described by the following equation:

3 $$ w^{\prime}= round(p * w) = \lfloor p*w + 0.5 \rfloor  $$

The proper repetitive sequence reconstruction requires high coverage $c = \frac {N*L}{G} \ge 100$. When *c*≥10 Poisson distribution of edge’s weight can be approximated by Normal distribution $\mathcal {N}(\mu, \sigma)$:

4 $$ W' \sim \mathcal{N}(\mu, \sigma)~\text{where}~\mu = \frac{\Delta}{d}, \sigma = \sqrt{\frac{\Delta}{d}}, \Delta = n-k+1  $$

For given level of confidence *q*, 0≤*q*≤1 we can calculate a required read coverage *c* for proper repetitive motif reconstruction, using the Eq. 5, where $\Phi _{N}^{-1}(q)$ is the inverse cumulative distribution function for standard normal distribution (*μ*=0, *σ*=1), *d* is the length of repetitive motif, *n* is the length of tandem repeats, *n*>*k*, *k* is de Bruijn graph dimension, *L* is read length.

5 $$ {}c = \frac{k}{L-k+1}\left(2 \Phi_{N}^{-1}\left(\frac{1+q}2\right)\right)^{2} \frac{\Delta}{d}~\text{where}~\Delta = n-k+1  $$

**Fig. 1:**
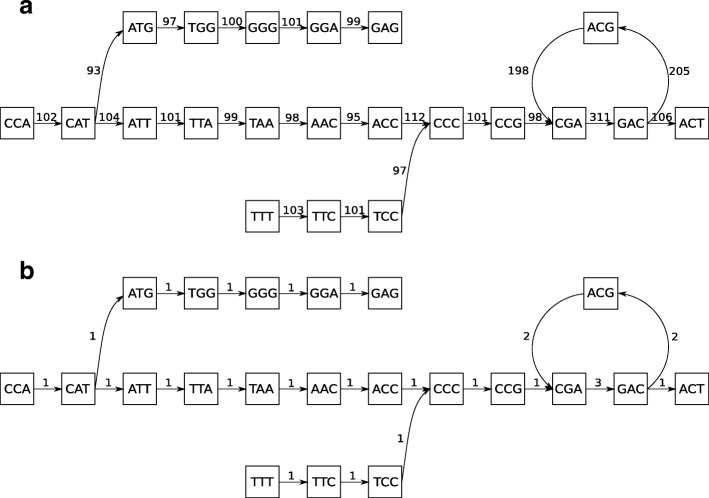
Edge weight normalisation. An example effect of edge weight normalisation. **a** Unnormalised graph – the edge weights depict the number of occurrences of a sequence of length k in a set of reads. **b** Normalised graph – the edge weights depict the number of occurrences of a DNA fragment of length k in the investigated genome

The process of estimating the number of occurrences of a given DNA fragment in the investigated genome is presented in Fig. 1.

#### Detecting tandem repeats

The next step of the tandem repeats reconstruction process is the detection of structures in the de Bruijn graph, which represent tandem repeats in the investigated genome. These structures appears as loops in de Bruijn graph connected with the rest of the graph by only one in-edge and only one out-edge. In other words, tandem repeats are represented by a sub-graph, where exactly one vertex has two in-edges and one out-edge, exactly one vertex has one in-edge and two out-edges, and all other vertices have one in-edge and one out-edge. Such structure consists of two parts:

- a branch from a vertex which represents an entry to the loop to a vertex which represents an exit of the loop;
- a branch from a vertex which represents an exit of the loop to a vertex which represents an entry to the loop.

**Fig. 2:**
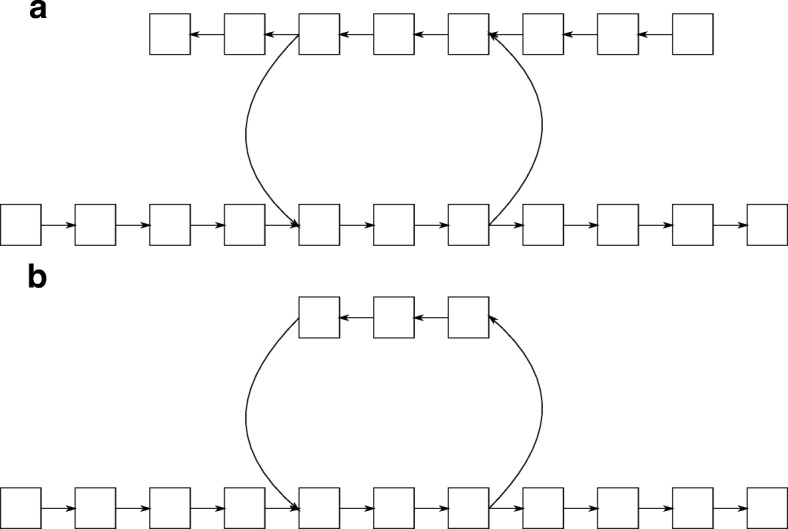
Tandem repeat detection. Sample structures representing and not representing tandem repeats in de Bruijn graph. **a** De Bruijn graph with an invalid tandem repeats structure – the loop in this example has two vertices with two in-edges and two vertices with two out-edges. **b** De Bruijn graph with a valid tandem repeats structure – the loop in this example has a single vertex with two in-edges and a single vertex with two out-edges

An example of a structure representing tandem repeat in the de Bruijn graph is presented in Fig. 2.

#### Correcting weights in tandem repeats

**Fig. 3:**
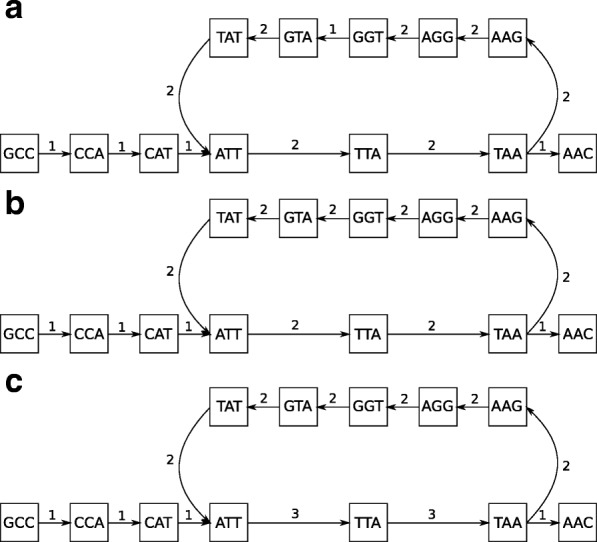
Correcting weights in loops of de Bruijn graph. A sample process of correcting weights in the de Bruijn graph. **a** The input de Bruijn graph. **b** De Bruijn graph after correcting weights in branches - the weight of (GGT,GTA) edge was changed to 2. The graph has uncorrected weights in valid loops - vertices ATT and TAA have degrees different from zero. **c** De Bruijn graph after correction weights in valid loops - the weights of less numerous branch (ATT,TTA,TAA) of the loop were changed so that all vertices of the loop will have degree equal to zero

The next step of the tandem repeats reconstruction process is the correction of the edge weights in the previously detected de Bruijn graph loops. Firstly, the weights in single branches are corrected so that all weights of the branch have the same weight. Secondly, the number of vertices in both parts of the loop are counted. Then, the edge weights in the less numerous parts of the loop are adapted to the weights of the edges of the more numerous parts of the loop, so that all of the vertices in the loop will be of 0 degree. Here, a degree is a sum of weights of vertex edges where the weights of in-edges are positive, and the weights of out-edges are negative. An example of correction of normalized edge weights in the de Bruijn graph loops is presented in Fig. 3.

#### Resolving tandem repeats in DNA sequence

The last step of reconstructing the repetitive DNA sequence from next-generation sequencing reads is to generate a DNA sequence from the de Bruijn graph. This process involves traverse the vertices of the de Bruijn graph until an ambiguous vertex is encountered.

The vertex is treated as unambiguous if it has zero, one or two input (output) edges and, in the case of exactly two input (output) edges, for one of them a simple return path exists ie. path from the target vertex to the source vertex, that has at least one vertex with more than one input edges and at least one vertex with more than one output edges. This condition makes the number of ambiguous vertices in our approach smaller than in the other existing assemblers, where ambiguity is set if a vertex has more than one input edge or more than one output edge.

The process of resolving tandem repeats consists of two steps: (1) finding vertices without any input edges and with at least one output edge, such vertex starts new contig and becomes current vertex; (2) iteratively processing directly connected vertices ie. adding them to actual contig and decrementing weights of visited edges; if the edge weight is 0, edge is removed from the graph. If the current vertex *v* is unambiguous, it extends the current contig, otherwise, it starts the new one. Moreover, if current vertex *v* is unambiguous and has two output edges, the edge, for which a previously defined simple return path exists, is chosen.

**Fig. 4:**
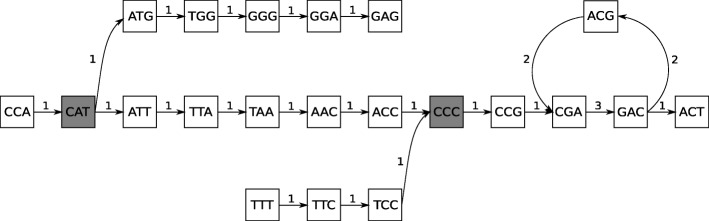
Generating DNA sequences from de Bruijn graph. An example process of building output contigs from a normalised de Bruijn graph. The resultant set of contigs should contain five contigs: CCAT, CATGGGAG, CATTAACCC, TTTCCC, and CCCGACGACGACT. The ambiguous vertices (CAT and CCC) are marked with gray colour

This process is repeated until all ambiguous vertices are resolved. An example of generating DNA sequences from de Bruijn graph is presented in Fig. 4.

#### Final assembly steps

All of the previously described steps of de novo assembly in dnaasm application lead to a generation set of DNA sequences called unitigs. Then, created unitigs could be extended to contigs and scaffolds using paired-end tags and mate-pairs - both algorithms are also implemented in dnaasm application.

### Implementation

**Fig. 5:**
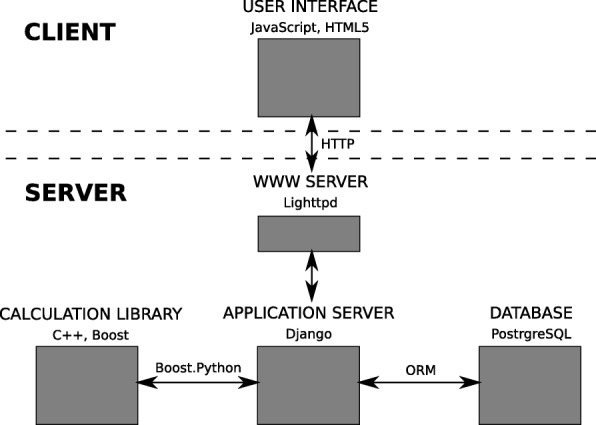
Modules of the dnaasm application. The main modules of dnaasm application. Dnaasm application could be treated as client-server application or command line software - the application could be run by the graphical user interface or by the command line. Moreover, command line version is developed in docker container, so that the installation is quick and easy

The web-application was developed in client-server architecture, where web-browser is used to communicate with end-user, Python is used to realize the application server, and algorithms are implemented in C++. The described architecture is based on a bioweb framework [11], the main modules of the application are presented in Fig. 5.

To achieve the high performance of calculation module we used several memory-efficient structures, e.g. Compressed Sparse Row Graph from Boost library to represent de Bruijn graph, Google Sparse Hash to implement hash map. Our advanced memory optimization enabled building and processing graph up to 7∗10^9^ vertices (e.g. for human genome) in 256 GB RAM. We deploy the module as shared C++ library.

## Results

In this section, we presented the results of tests for real data sets of bacterial organisms. We compared the results obtained by our approach with tandem repeats detected by algorithms based on paired-end tags. We also briefly describe new real assembly case from the whole genome sequencing project, where our approach gives an advantage. Moreover, we carried out several experiments on simulated datasets to compare efficiency of tandem repeats reconstruction.

### Comparison to another applications

We compared the dnaasm application with the three popular de novo DNA assemblers: ABySS [6] ver. 2.0.1, Velvet [7] ver. 1.2.10 and SPAdes [8] ver. 3.11.0. Applications were compared on four sets of bacterial DNA reads obtained from the National Center for Biotechnology Information. The benchmark dataset contains DNA reads from four samples - ERR351243 for *Helicobacter pylori PeCan4*, SRR5431732 for *Mycobacterium bovis*, SRR1981622 and SRR1981619 for *Helicobacter pylori J99*. The description of benchmark data sets is presented in Table 1.

**Table 1:** Sets of benchmark data

Sample	Coverage	Read length	Insert size mean	Insert size std dev
ERR351243	200x	100 bp	250 bp	63 bp
SRR1981622	200x	100 bp	226 bp	84 bp
SRR1981619	200x	100 bp	211 bp	78 bp
SRR5431732	150x	75 bp	268 bp	101 bp

To compare the performance of our approach and another de novo DNA assemblers we used four bacterial datasets - ERR351243, SRR1981622, SRR1981619 and SRR5431732. To speed up calculations, we decreased the value of genome coverage in ERR351243, SRR1981622 and SRR1981619 datasets to 200x

De novo assembling of the mentioned DNA reads was carried out in two modes - with and without using paired-ends tags. The results were compared in terms of the number of contigs longer than 1000 bp, the length of N50 contig, the length of the longest contig and two parameters describing the quality of the resultant sequences - the average number of mismatches and indels per 100,000 aligned bases. The above parameters were calculated by the quality assessment tool QUAST [12] ver. 4.1; and the results are presented in Table 2.

**Table 2:** Evaluation of dnaasm in comparison to ABySS, Velvet and SPAdes assembler

Sample	Assembler	Type	No. of	N50 [bp]	Max [bp]	Avg.	Avg.
		of output	contigs			mis.	indels
ERR351243	ABySS	unitigs	79	40021	224368	0.00	1.61
	Velvet	unitigs	206	12813	38818	0.31	0.81
	SPAdes	unitigs	43	61281	287932	0.93	2.28
	dnaasm	unitigs	84	30628	191880	0.06	1.98
	ABySS	scaffolds	33	65136	224368	1.59	2.69
	Velvet	scaffolds	30	82451	224509	1.42	3.15
	SPAdes	scaffolds	27	74784	333212	1.23	2.53
	dnaasm	scaffolds	34	61265	333846	0.62	3.82
SRR1981622	ABySS	unitigs	81	36448	110990	1.11	0.99
	Velvet	unitigs	254	8636	27708	1.12	0.37
	SPAdes	unitigs	53	58976	228504	1.42	1.29
	dnaasm	unitigs	73	45793	134195	0.93	1.11
	ABySS	scaffolds	40	67391	207675	4.89	1.96
	Velvet	scaffolds	33	114376	285632	4.99	2.65
	SPAdes	scaffolds	35	98439	228504	3.26	2.09
	dnaasm	scaffolds	39	91627	228504	3.33	1.73
SRR1981619	ABySS	unitigs	80	36449	110993	8.36	2.85
	Velvet	unitigs	314	7163	22826	7.92	1.56
	SPAdes	unitigs	53	58765	228586	9.00	3.21
	dnaasm	unitigs	77	43959	134261	7.76	2.77
	ABySS	scaffolds	43	79578	111309	16.00	4.15
	Velvet	scaffolds	35	107026	492812	10.06	4.32
	SPAdes	scaffolds	34	98351	228586	8.99	4.31
	dnaasm	scaffolds	38	93136	256270	11.21	4.25
SRR5431732	ABySS	unitigs	559	11471	57586	17.85	2.90
	Velvet	unitigs	1032	4790	18304	17.98	2.57
	SPAdes	unitigs	238	30607	129385	21.02	3.74
	dnaasm	unitigs	416	16178	59383	21.68	4.10
	ABySS	scaffolds	208	35552	131767	19.81	4.51
	Velvet	scaffolds	202	41843	182297	21.99	7.21
	SPAdes	scaffolds	172	46834	159097	22.65	4.72
	dnaasm	scaffolds	354	19644	120052	25.13	4.42

Unitigs output depicts assembling mode without using paired-end tags, scaffolds - with paired reads. The table shows that the presented de novo assembler works comparatively in terms of the number of contigs, N50 statistic, the largest contig length and the quality (average number of mismatches and indels per 100000 aligned bases) of the resultant DNA sequences

In Table 3 we showed the improvement of results by tandem repeat resolution. Furthermore, we counted the number of places in investigated samples, where our approach works properly and other assemblers fail. To compare the number of detected tandem repeats we used Tandem repeats finder application [13]; the results of this application are presented in Table 4.

**Table 3:** Evaluation of tandem repeats reconstruction algorithm in dnaasm

Sample	Type of output	With tandem repeats reconstruction			Without tandem repeats reconstruction		
		No. of contigs	N50 [bp]	Max [bp]	No. of contigs	N50 [bp]	Max [bp]
ERR351243	unitigs	84	30628	191880	93	27268	176501
	scaffolds	34	61265	333846	34	61265	333846
SRR1981622	unitigs	73	45793	134195	82	33787	110992
	scaffolds	39	91627	228504	43	80288	206366
SRR1981619	unitigs	77	43959	134261	81	36452	110995
	scaffolds	38	93136	256270	42	84434	205320
SRR5431732	unitigs	416	16178	59383	450	14774	47710
	scaffolds	354	19644	120052	388	17344	120052

Unitigs output depicts assembling mode without using paired-end tags, scaffolds - with paired reads. The table shows that tandem repeats reconstructuion process could significantly improve the results in terms of the number of contigs, N50 statistic and the largest contig length

**Table 4:** Detected tandem repeats in bacterial test datasets

Sample	Seq. len. [bp]	Motif len. [bp]	Min. cov. (Eq. 5)	Unitigs				Scaffolds			
				ABySS	Velvet	SPAdes	dnaasm	ABySS	Velvet	SPAdes	dnaasm
ERR351243	668	334	33	0	0	**2**	**2**	0	0	**2**	**2**
	371	16	371	0	0	0	0	0	0	0	0
SRR1981622	934	467	35	0	0	**2**	**2**	0	0	**2**	**2**
	706	307	39	0	0	0	0	0	0	0	0
	740	370	34	0	0	**2**	**2**	0	**2**	**2**	**2**
	1285	612	36	0	0	0	0	0	0	0	0
	1224	612	29	0	0	0	0	0	0	0	0
	1094	576	33	0	0	**1.9**	**1.9**	0	0	**1.9**	**1.9**
SRR1981619	934	467	35	0	0	**2**	**2**	0	0	**2**	**2**
	706	307	39	0	0	0	0	0	0	0	0
	740	370	34	0	0	**2**	**2**	0	0	**2**	**2**
	1285	612	36	0	0	0	0	0	0	0	0
	1224	612	29	0	0	0	0	0	0	0	0
	1094	576	33	0	0	**1.9**	**1.9**	0	0	**1.9**	**1.9**
SRR5431732	327	58	185	0	0	0	0	0	0	0	0
	335	69	160	0	0	0	0	0	0	0	0
	293	57	165	0	0	0	**5.1**	0	0	0	**5.1**
	267	51	164	0	0	0	**5.2**	0	0	0	**5.2**
	749	345	79	0	0	0	**2.2**	0	0	**2.2**	**2.2**
	579	111	186	0	0	0	**5.2**	0	0	0	**5.2**
	636	57	401	0	0	0	0	0	0	0	0

The numbers in the table depict number of motif repetitions in reconstructed DNA sequence. The proper restorations are in bold, the expected number of motif repetitions is defined as repetitive sequence length divided by the motif length. The table shows that only dnaasm and SPAdes reconstruct tandem repeats longer than insert size of paired-end tags in bacterial genomes. Moreover, dnaasm and SPAdes reconstruct these repetitive DNA regions even without using paired-end tags (unitigs). However, some of the tandem repeats are not reconstructed by any algorithm - they are contained in complex DNA regions, with many repeats of the same motif in other parts of the investigated genome

### Simulated reference genome

The next two experiments were carried out on the simulated data generated from the generated reference genome. This sequence consists of the 20 tandem repeats isolated from each other by a section of 1000 random symbols over {A, C, G, T} alphabet. The repetitive structures include: motif of length 100 bp repeated 2, 3, 4 and 5 times; motif of length 200 bp repeated 2, 3, 4 and 5 times; motif of length 300 bp repeated 2, 3, 4 and 5 times; motif of length 400 bp repeated 2, 3, 4 and 5 times; motif of length 500 bp repeated 2, 3, 4 and 5 times. The motifs were random symbols.

### Simulated dataset for different value of insert size

In this experiment we investigated how insert size affects the accuracy of tandem repeats detection. We generated sets of reads from simulated reference genome using the profile-based Illumina pair-end Read Simulator pIRS [14]. Three sets were generated:

- mean insert size: 250 bp, standard deviation of insert sizes: 25;
- mean insert size: 750 bp, standard deviation of insert sizes: 75;
- mean insert size: 1250 bp, standard deviation of insert sizes: 125.

The read length and depth of coverage for all simulated sets of reads was 100 bp and 150x, respectively. The substitution-error rate was 0.01, simulating indel errors in reads was switched on. To compare a number of detected tandem repeats we used Tandem repeats finder application [13]. The results are shown in Table 5.

**Table 5:** The efficiency of tandem repeats reconstruction from simulated data

Sequence len. [bp]	Motif len. [bp]	Unitigs				Scaffolds			
		ABySS	Velvet	SPAdes	dnaasm	ABySS	Velvet	SPAdes	dnaasm
200	100	0/0/0	0/0/0	**2**/**2**/**2**	**2**/**2**/**2**	**2**/**2**/**2**	**2**/**2**/**2**	**2**/**2**/**2**	**2**/**2**/**2**
300	100	0/0/0	0/0/0	0/0/0	**3**/**3**/**3**	2/**3**/**3**	0/2/0	2/2/2	**3**/**3**/**3**
400	100	0/0/0	0/0/0	0/0/0	**4**/**4**/**4**	2/**4**/**4**	0/0/0	2/2/2	**4**/**4**/**4**
500	100	0/0/0	0/0/0	0/0/0	**5**/**5**/6	2/0/**5**	0/0/0	2/2/2	**5**/**5**/6
400	200	0/0/0	0/0/0	**2**/**2**/**2**	**2**/**2**/**2**	0/**2**/**2**	**2**/**2**/**2**	**2**/**2**/**2**	**2**/**2**/**2**
600	200	0/0/0	0/0/0	0/0/0	**3**/**3**/**3**	0/0/0	0/2/2	2/2/2	**3**/**3**/**3**
800	200	0/0/0	0/0/0	0/0/0	**4**/**4**/**4**	0/0/0	0/0/2	2/2/2	**4**/**4**/**4**
1000	200	0/0/0	0/0/0	0/0/0	**5**/**5**/6	0/0/0	0/0/0	2/2/2	**5**/**5**/6
600	300	0/0/0	0/0/0	**2**/**2**/**2**	**2**/**2**/**2**	0/**2**/**2**	0/**2**/**2**	**2**/**2**/**2**	**2**/**2**/**2**
900	300	0/0/0	0/0/0	0/0/0	**3**/**3**/**3**	0/0/2	0/0/2	2/2/2	**3**/**3**/**3**
1200	300	0/0/0	0/0/0	0/0/0	**4**/**4**/**4**	0/0/0	0/0/0	2/2/2	**4**/**4**/**4**
1500	300	0/0/0	0/0/0	0/0/0	**5**/**5**/**5**	0/0/0	0/0/0	2/2/2	**5**/**5**/**5**
800	400	0/0/0	0/0/0	**2**/**2**/**2**	**2**/**2**/**2**	0/**2**/0	0/**2**/**2**	**2**/**2**/**2**	**2**/**2**/**2**
1200	400	0/0/0	0/0/0	0/0/0	**3**/**3**/**3**	0/2/0	0/0/0	0/2/2	**3**/**3**/**3**
1600	400	0/0/0	0/0/0	0/0/0	**4**/**4**/**4**	0/2/0	0/0/0	0/2/2	**4**/**4**/**4**
2000	400	0/0/0	0/0/0	0/0/0	**5**/**5**/**5**	0/0/0	0/0/0	0/2/2	**5**/**5**/**5**
1000	500	0/0/0	0/0/0	**2**/**2**/**2**	**2**/**2**/**2**	0/**2**/**2**	0/**2**/**2**	**2**/**2**/**2**	**2**/**2**/**2**
1500	500	0/0/0	0/0/0	0/0/0	**3**/**3**/**3**	0/2/0	0/0/0	0/2/2	**3**/**3**/**3**
2000	500	0/0/0	0/0/0	0/0/0	**4**/**4**/**4**	0/2/0	0/0/0	0/2/2	**4**/**4**/**4**
2500	500	0/0/0	0/0/0	0/0/0	**5**/**5**/**5**	0/0/0	0/0/0	0/2/2	**5**/**5**/**5**

The numbers in the table depict number of motif repetitions in reconstructed DNA sequence for insert size mean equal to 250 bp, 750 bp and 1250 bp, respectively. The proper restorations are in bold, the expected number of motif repetitions is defined as in Table 4. Only dnaasm was able to reconstruct tandem repeats with more than two motif repetition from unitigs. Additionally, the ABySS results of 100 bp motif reconstruction from paired-end tags shows, that increasing the insert size value increases the probability of tandem repeat reconstruction

### Simulated dataset for different depth of coverage

In this experiment we checked how read coverage affects the tandem repeats detection for different types of repetitive sequences - we compared efficiency of reconstructing tandem repeats by our approach and by methods based on paired-end tags on simulated datasets generated with another depth of coverage. We used, as in the previous experiment, dataset generated by read simulator from our reference genome, The read length, insert size mean and standard deviation of insert sizes was 100 bp, 250 bp and 25, respectively, the error simulation parameters – as in previous experiment. We generated three sets of input paired-end tags with depth of coverage: 50x, 100x and 150x. The results are depicted in Table 6.

**Table 6:** The efficiency of tandem repeats reconstruction from simulated data

Sequence len. [bp]	Motif len. [bp]	Min. cov. (Eq. 5)	Unitigs				Scaffolds			
			ABySS	Velvet	SPAdes	dnaasm	ABySS	Velvet	SPAdes	dnaasm
200	100	26	0/0/0	0/0/0	**2**/**2**/**2**	**2**/**2**/**2**	**2**/0/**2**	**2**/**2**/**2**	**2**/**2**/**2**	**2**/**2**/**2**
300	100	44	0/0/0	0/0/0	0/0/0	4/**3**/**3**	0/0/2	0/0/0	2/2/2	4/**3**/**3**
400	100	62	0/0/0	0/0/0	0/0/0	4/**4**/**4**	0/0/2	0/0/0	2/2/2	4/**4**/**4**
500	100	80	0/0/0	0/0/0	0/0/0	6/6/**5**	0/0/2	0/0/0	2/2/2	6/6/**5**
400	200	31	0/0/0	0/0/0	**2**/**2**/**2**	**2**/**2**/**2**	0/0/0	**2**/**2**/**2**	**2**/**2**/**2**	**2**/**2**/**2**
600	200	49	0/0/0	0/0/0	0/0/0	4/**3**/**3**	0/0/0	0/0/0	2/2/2	4/**3**/**3**
800	200	66	0/0/0	0/0/0	0/0/0	3/**4**/**4**	0/0/0	0/0/0	2/2/2	3/**4**/**4**
1000	200	84	0/0/0	0/0/0	0/0/0	6/6/**5**	0/0/0	0/0/0	2/2/2	6/6/**5**
600	300	32	0/0/0	0/0/0	**2**/**2**/**2**	**2**/**2**/**2**	0/0/0	0/0/0	**2**/**2**/**2**	**2**/**2**/**2**
900	300	50	0/0/0	0/0/0	0/0/0	**3**/**3**/**3**	0/0/0	0/0/0	2/2/2	**3**/**3**/**3**
1200	300	68	0/0/0	0/0/0	0/0/0	**4**/**4**/**4**	0/0/0	0/0/0	2/2/2	**4**/**4**/**4**
1500	300	86	0/0/0	0/0/0	0/0/0	**5**/**5**/**5**	0/0/0	0/0/0	2/2/2	**5**/**5**/**5**
800	400	33	0/0/0	0/0/0	**2**/**2**/**2**	**2**/**2**/**2**	0/0/0	0/0/0	**2**/**2**/**2**	**2**/**2**/**2**
1200	400	51	0/0/0	0/0/0	0/0/0	**3**/**3**/**3**	0/0/0	0/0/0	0/0/0	**3**/**3**/**3**
1600	400	69	0/0/0	0/0/0	0/0/0	**4**/**4**/**4**	0/0/0	0/0/0	0/0/0	**4**/**4**/**4**
2000	400	87	0/0/0	0/0/0	0/0/0	**5**/**5**/**5**	0/0/0	0/0/0	0/0/0	**5**/**5**/**5**
1000	500	33	0/0/0	0/0/0	**2**/**2**/**2**	**2**/**2**/**2**	0/0/0	0/0/0	**2**/**2**/**2**	**2**/**2**/**2**
1500	500	51	0/0/0	0/0/0	0/0/0	**3**/**3**/**3**	0/0/0	0/0/0	0/0/0	**3**/**3**/**3**
2000	500	69	0/0/0	0/0/0	0/0/0	**4**/**4**/**4**	0/0/0	0/0/0	0/0/0	**4**/**4**/**4**
2500	500	87	0/0/0	0/0/0	0/0/0	**5**/**5**/**5**	0/0/0	0/0/0	0/0/0	**5**/**5**/**5**

The numbers in the table depict number of motif repetitions in reconstructed DNA sequence for depth of coverage 50x, 100x and 150x, respectively. The proper restorations are in bold, the expected number of motif repetitions is defined as in Table 4. Only our algorithm was able to reconstruct tandem repeats with more than two motif repetition. It is worth paying attention to dnaasm results for 100 bp and 200 bp motifs, where increasing the depth of coverage increases the probability of tandem repeat correct reconstruction

### PCR confirmation

To present the correctness and usefulness of our approach, we use our application in a project managed by the Witold Stefański Institute of Parasitology of the Polish Academy of Sciences dealing with, inter alia, the problem of sequencing and assembling mitochondrial DNA of rat tapeworm *Hymenolepis diminuta*. Despite the small size of this sequence (only 13,900 bp), there is a large repetitive DNA region (tandem repeats), which contains 13 repeats of the same 31-nt sequence [15]. To assemble this sequence, we obtained reads from the Illumina sequencer, the reads were paired (2x100 bp), an average insert size was equal to 300 bp. Unfortunately, the insert size of paired-end tags was smaller than the length of the investigated repetitive region. Due to this fact, our application, as the only one DNA assembler, was able to reconstruct this repetitive region. Moreover, the depth of coverage for this sequencing project was high, ie. for mitochondrial DNA above 1000x, so we were able to use our application several times with different coverage depths (from 300x to 1000x). The results for all these calculations were the same, especially, the DNA fragment with tandem repeat was always reconstructed. What is more, additional ultra-deep sequencing of PCR amplicons for this DNA region confirmed the results obtained by our approach.

## Discussion

In this paper we describe an application used to reconstruct some of the repetitive DNA regions based on the normalised read depth. The presented approach was thoroughly tested and the experiments carried out on the simulated data, described in this paper, confirmed our concept. What is more, the reconstruction of repetitive DNA region was proved by biological experiments.

The read coverage of the genome region is key to the correct reconstruction of the repetitive fragment in our approach. However, the read depth of the specific DNA region varies depending on the GC content [16]. There are many methods for correction of the GC bias [17], most of them are implemented in copy number variation (CNV) detection tools based on read depth. Implementation and testing of some correction GC bias algorithm in our approach is one of the most important tasks in the near future.

Nowadays, nanopore sequencers are very popular. They allow to obtain the DNA reads of length greater than 10 kbp. The main disadvantage of nanopore sequencing is that obtained data contains more errors than the second generation sequencing reads. However, the usage of the long reads can improve the assembly results from the short reads [18]. The presented algorithm currently does not use long reads. However, we plan to integrate such sequencing data in the next version of the software.

What is more, in the future we plan to add the possibility of running the application on a computer cluster. The de novo assembler will be divided into the set of containers, which will be managed and run by Apache Spark. The new architecture will allow to disperse the calculation, which will significantly reduce the time of de novo assembling. Furthermore, in the future we plan to create a virtual machine [19] image and an Amazon machine image.

The demo application with web interface as well as source code of the application are available at project homepage^1^. What is more, there is a public Docker container [20] with dnaasm de novo assembler. The presented application is freely available to both academic and commercial users under GNU Library or Lesser General Public License version 3.0 (LGPLv3).

## Conclusions

As more and more bacterial genomes are sequenced, it becomes desirable to analyze their tandem repeats. Here we have presented dnaasm, a de novo DNA assembler that uses the relative frequency of reads to properly reconstruct repetitive sequences, especially, in bacterial genomes.

## Footnotes

1
http://dnaasm.sourceforge.net
